# Secondary Metabolites from *Hericium erinaceus* and Their Anti-Inflammatory Activities

**DOI:** 10.3390/molecules27072157

**Published:** 2022-03-27

**Authors:** Guangbo Xie, Lan Tang, Yu Xie, Liyuan Xie

**Affiliations:** 1 School of Life Science and Technology, University of Electronic Science and Technology of China, Chengdu 610054, China; 201821140607@std.uestc.edu.cn (L.T.); 201921140426@std.uestc.edu.cn (Y.X.); 2 Sichuan Institute of Edible Fungi, Sichuan Academy of Agricultural Sciences, Chengdu 610066, China

**Keywords:** *Hericium erinaceus*, secondary metabolites, isolation and structural elucidation, anti-inflammatory activity

## Abstract

*Hericium erinaceus*, a culinary and medicinal mushroom, is widely consumed in Asian countries. Chemical investigation on the fruiting bodies of *Hericium erinaceus* led to the isolation of one new ergostane-type sterol fatty acid ester, erinarol K (**1**); and eleven known compounds: 5α,8α -epidioxyergosta-6,22-dien-3β-yl linoleate (**2**); ethyl linoleate (**3**); linoleic acid (**4**); hericene A (**5**); hericene D (**6**); hericene E (**7**); ergosta-4,6,8(14),22-tetraen-3-one (**8**); hericenone F (**9**); ergosterol (**10**); ergosterol peroxide (**11**); 3β,5α,6α,22*E*-ergosta-7,22-diene-3,5,6-triol 6-oleate (**12**). The chemical structures of the compounds were determined by 1D and 2D NMR (nuclear magnetic resonance) spectroscopy, mass spectra, etc. Anti-inflammatory effects of the isolated aromatic compounds (**5**–**7**, **9**) were evaluated in terms of inhibition of pro-inflammatory mediator (TNF-α, IL-6 and NO) production in lipopolysaccharide (LPS)-stimulated murine RAW 264.7 macrophage cells. The results showed that compounds **5** and **9** exhibited moderate activity against TNF-α (IC_50_: 78.50 μM and 62.46 μM), IL-6 (IC_50_: 56.33 μM and 48.50 μM) and NO (IC_50_: 87.31 μM and 76.16 μM) secretion. These results supply new information about the secondary metabolites of *Hericium erinaceus* and their anti-inflammatory effects.

## 1. Introduction

Mushrooms are familiar food ingredients and frequently appear on the daily dining table. Their wide consumption is not only due to their unique flavor and texture as an attractive food, but also to their beneficial effects on human health. *Hericium erinaceus* (Bull.) Pers. (family Hericiaceae), also known as Houtougu (monkey head) in Chinese, Lion’s Mane in English and Yamabushitake in Japanese after its shape, is a popular edible and medicinal mushroom widely consumed in Asian countries (China, Japan and Korea, etc.) [1]. *H. erinaceus* grows on old or dead trunks of hard woods and its fruiting bodies have been used in traditional Chinese medicine for treatment of gastritis for more than 1000 years [2]. Recently, the beneficial effects of the fruiting bodies of *H. erinaceus* on depression, anxiety and cognitive impairment were also reported [3,4]. Previous chemical investigations on *H. erinaceus* have established the presence of an exceptionally large amount of structurally different bioactive and potential bioactive components, such as diterpenoids (erinacines) [5], aromatic compounds (hericerins, erinacerins and erinaceolactones) [6,7,8,9,10], sterols [11,12], polysaccharides and glycoproteins [13,14,15]. These isolated components of *H. erinaceus* were reported to possess various bioactivities, such as cytotoxicity [9,10], immunomodulation [16,17], nerve growth factor (NGF) promotion [18,19], and antidiabetic [7,8] properties.

**Figure 1:**
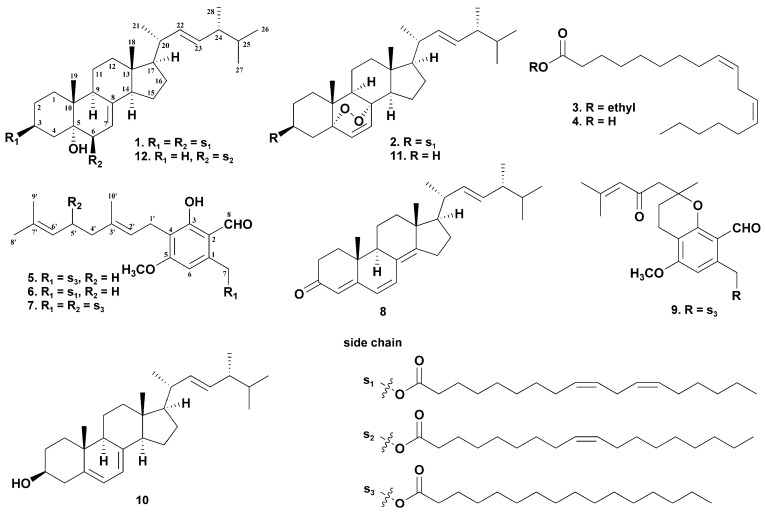
Chemical structures of compounds **1**–**12**.

In our continuing investigation on edible and medicinal mushrooms [20,21,22,23], one new (**1**) and eleven known compounds (**2**–**12**, Figure 1) were isolated from the fruiting bodies of *H. erinaceus*. Here, we report the structural elucidation of the isolated components and the anti-inflammatory effects of the isolated aromatic compounds.

## 2. Results and Discussion

**Figure 2:**
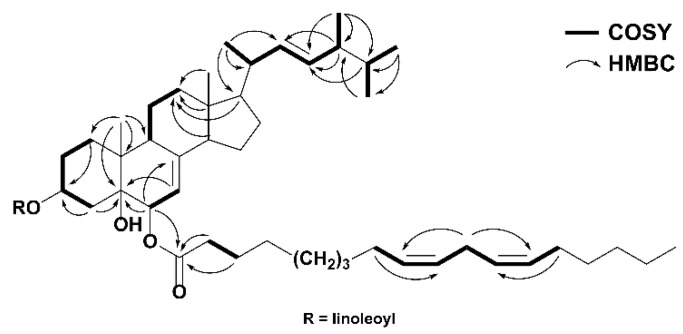
^1^H-^1^H COSY and key HMBC correlations of **1**. (COSY: correlation spectroscopy).

**Figure 3:**
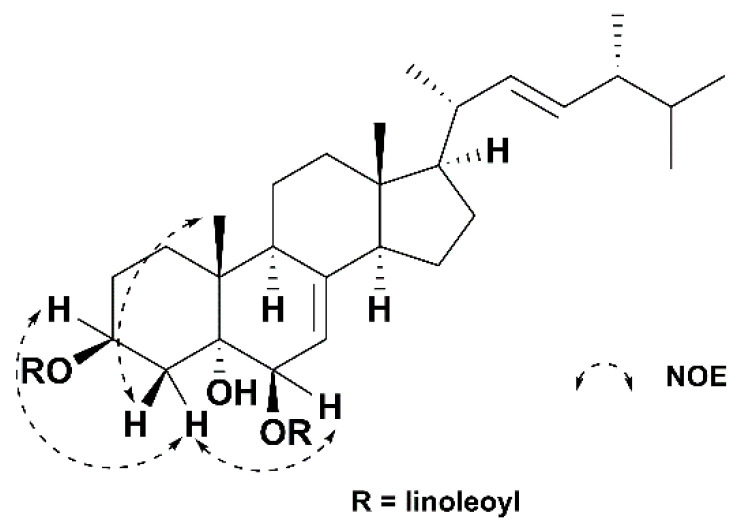
NOESY correlations of **1**.

Compound **1** was isolated as a colorless, oily solid. Its molecular formula was established as C_64_H_106_O_5_ using HRESIMS (high resolution electrospray ionization mass spectrometry) (*m*/*z* 955.8112 [M + H]^+^; calcd 955.8119), indicating 12 degrees of unsaturation. The IR spectrum indicated the presence of hydroxyl group (3435 cm^−1^) and carbonyl group (1736 cm^−1^). The ^1^H NMR data of 1 (Table 1) showed six methyls [δ_H_ 0.57 (s, Me-18), 0.82 (d, *J* = 6.4 Hz, Me-27), 0.83 (d, *J* = 6.4 Hz, Me-26), 0.91 (d, *J* = 6.8 Hz, Me-28), 1.02 (d, *J* = 6.5 Hz, Me-21), 1.06 (s, Me-19)], two oxymethines [δ_H_ 4.80 (d, *J* = 4.7 Hz, H-6α), 5.14 (m, H-3α)], and three olefinic protons [δ_H_ 5.19 (m, H-22), 5.21 (m, H-23), 5.28 (m, H-7)]. Additionally, two typical linoleic acid residues were observed, including eight olefinic protons [δ_H_ 5.30–5.40 (H-9′, 9″, 10′, 10″, 12′, 12″, 13′, 13″)], twenty-four methylenes [δ_H_ 1.23–2.03 (m, H-3′-8′, 14′-17′, 3″-8″, 14″-17″), 2.25 (t, *J* = 7.5 Hz, H_a_-2′/2″), 2.30 (t, *J* = 7.2 Hz, H_b_-2′/2″), 2.77 (t, *J* = 6.3 Hz, H-11′ and 11″)], and two terminal methyls [δ_H_ 0.88 (H-18′ and 18″)]. Its ^13^C NMR spectrum (Table 1) showed 28 resonances of the sterol moiety, including six methyls [δ_C_ 12.3 (C-18), 17.6 (C-28), 18.2 (C-19), 19.6 (C-27), 19.9 (C-26), 21.1 (C-21)], seven sp^3^ methylenes [δ_C_ 21.9 (C-11), 22.8 (C-15), 26.9 (C-2), 27.8 (C-16), 32.2 (C-1), 35.7 (C-4), 39.1 (C-12)], eight sp^3^ [two oxygenated at δ_C_ 70.4 (C-3), 73.4 (C-6)] and three sp^2^ [δ_C_ 114.0 (C-7), 132.1 (C-23), 135.3 (C-22)] methines, and three sp^3^ [one oxygenated at δ_C_ 74.9 (C-5)] and one sp^2^ [δ_C_ 145.6 (C-8)] quaternary carbons. Signals of two linoleic acid residues were also observed in the ^13^C NMR spectrum, showing eight olefinic carbons [δ_C_ 127.8, 127.9, 18.0, 128.0 (C-9′, 9″, 10′, 10″), 130.0, 130.0, 130.2, 130.2 (C-12′, 12″, 13′, 13″)], twenty-four methylene groups [δ_C_ 22.5–34.6 (C-2′-8′, C-2″-8″, C-11′, C-11″, C-14′-17′, C-14″-17″)], two methyl groups [δ_C_ 14.0, 14.1 (C-18′, 18″)], and two carbonyl groups [δ_C_ 173.0 (C-1″), 173.3 (C-1′)]. The data above indicates the sterol moiety of **1** was a Δ^7,8^ ergostane derivative, closely resembling those of (22*E*,24*R*)-ergosta-7,22-diene-3β,5α,6β-triol, a known sterol previously isolated from the fruiting bodies of *H. erinaceum* [24]. The only difference between these two compounds is that 1 had two additional linoleic acid residues at C-3 and C-6, which were supported by the key HMBC (heteronuclear multiple bond correlation) correlations between H-3 (δ_H_ 5.14) and C-1′ (δ_C_ 173.3), H-6 (δ_H_ 4.80) and C-1″ (δ_C_ 173.0) (Figure 2). The relative configuration of **1** was determined using a NOESY (nuclear overhauser effect spectroscopy) NMR experiment (Figure 3). Therefore, compound **1** was determined to be (22*E*,24*R*)-ergosta-7,22-diene-3β,5α,6β-triol 3,6-dilinoleate, and named erinarol K.

The structures of the eleven known compounds were identified by comparing HRESIMS, ^1^H NMR and ^13^C NMR data with the literature, as 5α,8α-epidioxyergosta-6,22-dien-3β-yl linoleate (**2**) [25], ethyl linoleate (**3**) [26], linoleic acid (**4**) [27], hericene A (**5**) [7], hericene D (**6**) [10], hericene E (**7**) [8], ergosta-4,6,8(14),22-tetraen-3-one (**8**) [28], hericenone F (**9**) [29], ergosterol (**10**) [30], ergosterol peroxide (**11**) [31], 3β,5α,6α,22*E*-ergosta-7,22-diene-3,5,6-triol 6-oleate (**12**) [32].

TNF-α, IL-6 and NO, the major pro-inflammatory mediators, are able to induce inflammation due to overproduction in abnormal situations [33,34,35], and the inhibition effects on their secretion are often used in evaluating the potential anti-inflammatory activities of the isolated natural products [12,36]. Bacterial lipopolysaccharide (LPS) is the best characterized stimulus for the induction of inflammatory mediators in macrophage RAW 264.7 [37]. On the basis of the traditional use in treating gastritis by *H. erinaceus* [2], we evaluated the potential anti-inflammatory activity of hericene A, D and E (**5**–**7**), hericenone F (**9**), one type of characteristic aromatic compound only isolated from *H. erinaceus*, using LPS-stimulated RAW 264.7 mouse cells as the cell model.

**Figure 4:**
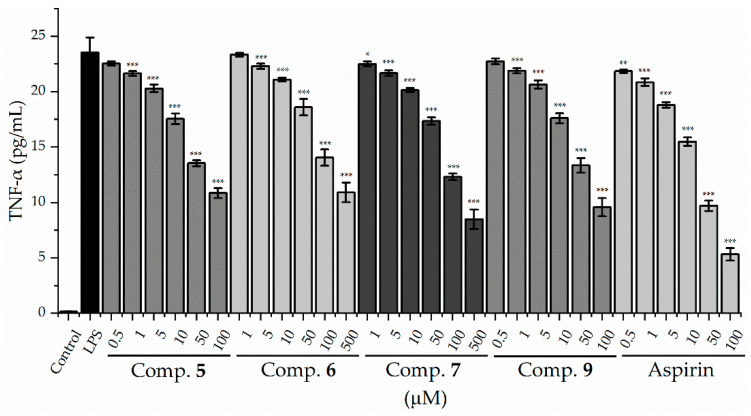
Effects of compounds **5**–**7** and **9** on TNF-α production in LPS-stimulated raw 264.7 cells. * *p* < 0.05, ** *p* < 0.01, and *** *p* < 0.001 compared with LPS-stimulated group. Aspirin (positive control).

**Figure 5:**
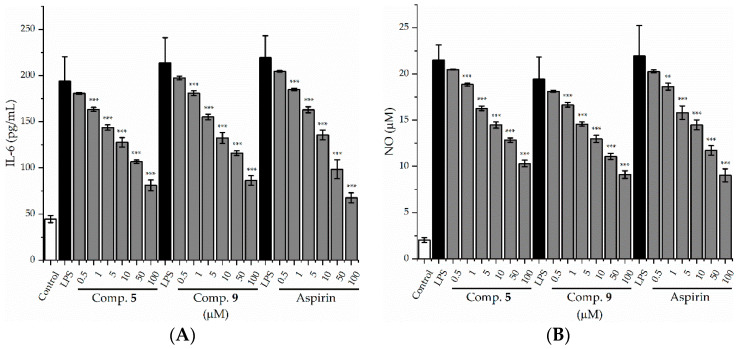
Effects of compounds **5** and **9** on IL-6 (**A**) and NO (**B**) production in LPS-stimulated raw 264.7 cells. ** *p* < 0.01, and *** *p* < 0.001 compared with LPS-stimulated group. Aspirin (positive control).

First, cell viability was evaluated using the CCK-8 assay. The results showed that compounds **5**–**7** and **9** did not affect cell viability at the tested concentrations. As shown in Figure 4, the secretion of TNF-α was significantly inhibited by compounds **5**–**7** and **9** in a dose-dependent manner, and compounds **5** and **9** showed the most potent inhibitory activities on the production of inflammatory factor TNF-α, with IC_50_ values of 78.50 and 62.46 μM, respectively, compared with the positive control (Aspirin, IC_50_ 27.08 μM) (Table 2). We, therefore, further evaluated compounds **5** and **9** for their inhibition on the secretion of IL-6 and NO, another two pro-inflammatory mediators, in LPS-stimulated RAW 264.7 mouse cells. As shown in Figure 5 and Table 2, compounds **5** and **9** also inhibited the secretion of IL-6 and NO in a dose-dependent manner, with IC_50_ values of 56.33 and 48.5 μM (IL-6), 87.31 and 76.16 μM (NO), respectively, compared with aspirin (IC_50_, 28.43 μM for IL-6; 51.82 μM for NO).

## 3. Materials and Methods

### 3.1. General Experimental Procedures

Optical rotation was measured using a Rudolph Research Analytical APVI/6W automatic polarimeter (Hackettstown, NJ, USA). The FT-IR spectrum was recorded on a ThermoFisher Nicolet 6700 FT-IR spectrometer (Waltham, MA, USA). The NMR spectra were recorded using Bruker AV II 600 and 400 (Billerica, MA, USA), with tetramethylsilane as an internal standard. The high-resolution electrospray ionization mass spectra (HRESIMS) were obtained using a Water Q-TOF Premier (Milford, MA, USA). Column chromatography was performed using silica gel (200–300 mesh, Qingdao Marine Chemical Company, Qingdao, China) and Sephedex LH-20 (GE Healthcare Bio-Sciences AB, Uppsala, Sweden); thin-layer chromatography (TLC) was performed using precoated silica gel GF_254_ (0.2–0.25 mm, Qingdao Haiyang Chemical Co., Qingdao, China).

### 3.2. Fungal Material

Mature fruiting bodies of *H. erinaceus* were collected from a planting base in Jintang District, Chengdu, China, in September 2018 and identified by one of the authors (L.X.). A voucher specimen (HE-201809) was deposited at the Sichuan Institute of Edible Fungi, Sichuan Academy of Agricultural Sciences.

### 3.3. Extraction and Isolation

Oven-dried fruiting bodies (10 kg) of *H. erinaceus* were extracted with 95% EtOH (45 L × 3) under room temperature (7d each time). The EtOH extract was concentrated in vacuo to yield a residue (2.1 L), which was further suspended in water and partitioned with EtOAc (6 L × 3), yielding EtOAc fractions (125 g).

The EtOAc fraction (125 g) was subjected to column chromatography over silica gel (200–300 mesh, 1.8 kg) and eluted with petroleum ether-EtOAc (120:1–1:1, gradient system) to yield eighteen fractions (Fr. 1–18). The fraction Fr. 3 (3 g) was separated using silica gel column chromatography with a gradient of cyclohexane-EtOAc (100:1 to 10:1) to yield 5 subfractions (Fr. 3-1–3-5). The fraction Fr. 3-2 was isolated by silica gel column chromatography (cyclohexane-EtOAc, 45:1) and then purified by Sephadex LH-20 column chromatography (CHCl_3_-MeOH, 2:1) to yield compound 2 (20 mg). The fraction Fr. 3-5 was subjected to silica gel column chromatography (cyclohexane-EtOAc, 80:1) and then purified by Sephadex LH-20 column chromatography (CHCl_3_-MeOH, 2:1) to yield compound 3 (8 mg). The fraction Fr. 5 (0.8 g) was separated using silica gel column chromatography (petroleum ether-EtOAc, 65:1) to yield 5 subfractions (Fr. 5-1–5-5). The fraction Fr. 5-3 was purified by Sephadex LH-20 column chromatography (CHCl_3_-MeOH, 2:1) to yield compound 4 (10 mg). The fraction Fr. 6 (0.82 g) was separated using silica gel column chromatography with a gradient of petroleum ether-EtOAc (60:1 to 1:1) to yield 6 subfractions (Fr. 6-1–6-6). The fraction Fr. 6-2 was purified by Sephadex LH-20 column chromatography (CHCl_3_-MeOH, 2:1) to yield compound 5 (50 mg). The fraction Fr. 8 (0.75 g) was separated using silica gel column chromatography (cyclohexane-EtOAc, 40:1) to yield 5 subfractions (Fr. 8-1–8-5). The fraction Fr. 8-3 was purified by Sephadex LH-20 column chromatography (CHCl_3_-MeOH, 2:1) to yield compound 6 (10 mg). The fraction Fr. 9 (0.62 g) was separated using silica gel column chromatography with a gradient of petroleum ether-EtOAc (80:1 to 1:1) to yield 7 subfractions (Fr. 9-1–9-7). The fraction Fr. 9-5 was purified by Sephadex LH-20 column chromatography (CHCl_3_-MeOH, 2:1) to yield compound 7 (10 mg). The fraction Fr. 9-6 was subjected to silica gel column chromatography (petroleum ether-EtOAc, 40:1) and then purified by Sephadex LH-20 column chromatography (CHCl_3_-MeOH, 2:1) to yield compound 1 (10 mg). The fraction Fr. 10 (0.6 g) was separated using silica gel column chromatography (petroleum ether-EtOAc, 60:1) to yield 6 subfractions (Fr. 10-1–10-6). The fraction Fr. 10-3 was subjected to silica gel column chromatography (petroleum ether-EtOAc, 40:1) and then purified by Sephadex LH-20 column chromatography (CHCl_3_-MeOH, 2:1) to yield compound 8 (8 mg). The fraction Fr. 11 (3.2 g) was separated using silica gel column chromatography (petroleum ether-EtOAc, 60:1) to yield 7 subfractions (Fr. 11-1–11-7). The fraction Fr. 11-4 was subjected to silica gel column chromatography (petroleum ether-EtOAc, 30:1) and then purified by Sephadex LH-20 column chromatography (CHCl_3_-MeOH, 2:1) to yield compound 9 (30 mg). The insoluble part of Fr. 12 was recrystallized using EtOAc to yield compound 10 (300 mg). The fraction Fr. 13 (2.1 g) was separated using silica gel column chromatography (petroleum ether-EtOAc, 50:1) to yield 5 subfractions (Fr. 13-1–13-5). The fraction Fr. 13-5 was subjected to silica gel column chromatography (cyclohexane-EtOAc, 20:1) and then purified by Sephadex LH-20 column chromatography (CHCl_3_-MeOH, 2:1) to yield compound 11 (11 mg). The fraction Fr. 15 (1.3 g) was separated using silica gel column chromatography (petroleum ether-EtOAc, 40:1) to yield 5 subfractions (Fr. 15-1–15-5). The fraction Fr. 15-3 was subjected to silica gel column chromatography (petroleum ether-EtOAc, 20:1) and then purified by Sephadex LH-20 column chromatography (CHCl_3_-MeOH, 2:1) to yield compound 12 (18 mg).

*Erinarol K* (**1**). Colorless oily solid; [α]21D −35.9 (*c* 2.08 × 10^−3^, CHCl_3_); IR (KBr) ν_max_ 3435, 2925, 2854, 1736, 1461, 1377, 1259, 1168, 758 cm^−1^; ^1^H and ^13^C NMR spectroscopic data, see Table 1; HRESIMS *m*/*z* 955.8112 [M + H]^+^ (calcd for C_64_H_107_O_5_^+^, 955.8119).

### 3.4. Anti-Inflammatory Activity Assay

#### 3.4.1. Cell Culture

Raw 264.7 mouse cells (ATCC, Rockville, MD, USA) were cultured in RPMI 1640 (GIBCO, Invitrogen Corporation, Carlsbad, CA, USA) supplemented with 10% fetal bovine serum (FBS) (GIBCO, Invitrogen Corporation, Carlsbad, CA, USA), 100 units/mL penicillin and 100 μg/mL streptomycin (all from Sigma, St. Louis, MO, USA), and then cultured in an incubator at 37 °C under a 5% CO_2_ atmosphere. Compounds were dissolved in chloroform to make stock solutions of 10 mM (compounds **5**, **9** and aspirin) and 50 mM (compounds **6** and **7**), which were then diluted in culture medium to obtain the desired concentrations. Aspirin was used as the positive control.

#### 3.4.2. Cell Viability

Cell viability was evaluated by the CCK-8 (Cell Counting Kit-8) method. Compounds in different concentration were added to the cells and incubated for 2 h, and then CCK-8 solution (10 μL, Beyotime, Shanghai, China) was added. The cells were further incubated for 4 h and then the absorbance was measured at 450 nm.

#### 3.4.3. Pro-Inflammatory Cytokines (TNF-α and IL-6) Assay

The production of TNF-a and IL-6 was measured according to the literature with minor modification [38]. RAW 264.7 cells were cultured at a density of 1 × 10^5^ cells/well in RPMI 1640. Cells were pretreated with different concentrations of compounds for 2 h before LPS stimulation. Twenty-four hours after LPS (200 ng/mL) stimulation, TNF-α (TNF-α Elisa kit, Boster Biological Technology Co. Ltd., Wuhan, China) and IL-6 (Mouse IL-6 Elisa kit, Beyotime, Shanghai, China) levels in the supernatant were measured by the ELISA test according to the manufacturer’s instructions.

#### 3.4.4. Nitric Oxide (NO) Assay

The production of NO was measured using the Griess method as previously reported with minor modification [39]. Briefly, the RAW 264.7 cells were pretreated with different concentrations of compounds for 2 h before LPS stimulation. Twenty-four hours after LPS (200 ng/mL) stimulation, 50 μL Griess reagent I and 50 μL Griess reagent II (Beyotime, Shanghai, China) were added into the 50 μL supernatant, respectively. This mixture was incubated for 10 min at room temperature, and the absorbance was measured at 540 nm using a microplate reader (LB 941, Berthold Technologies, Bad Wildbad, Germany). The amount of nitrite in the samples was obtained by a calibration curve using NaNO_2_ as the standard.

#### 3.4.5. Statistical Analysis

GraphPad Prism 6 (GraphPad Software Inc., San Diego, CA, USA) was used for data processing and analysis. The data obtained are presented as the means ± SD of five independent experiments. A one-way analysis of variance (ANOVA) followed by Tukey’s test was used to determine significant differences between each treated group and the LPS group. Values of *p* < 0.05 (*), *p* < 0.01 (**) and *p* < 0.001 (***) were considered to indicate statistical significance.

## 4. Conclusions

In this study, the chemical constitution of the fruiting bodies of *H. erincacus* was studied, and twelve compounds, including one new compound and eleven known compounds were isolated. The four typical aromatic compounds were evaluated for their inhibition effects on the secretion of TNF-α, IL-6 and NO, three major pro-inflammatory mediators, in the macrophage RAW 264.7 model. Two of them showed moderate inhibitory effects indicating their potential anti-inflammatory activity, which may provide the basis for the traditional medical use of *H. erincacus*.

## Figures and Tables

**Table 1:** ^1^H NMR (400 MHz) and ^13^C NMR (100 MHz) Spectroscopic Data for **1** in CDCl_3_.

Position	δ_C_	δ_H_ (*J* in Hz)	Position	δ_C_	δ_H_ (*J* in Hz)
1	32.2	1.55 (m); 1.70 (m)	24	42.8	1.84 (m)
2	26.9	1.49 (m); 1.89 (m)	25	33.0	1.46 (m)
3	70.4	5.14 (m)	26	19.9	0.83 (d, *J* = 6.48)
4	35.7	1.71 (m); 1.96 (m)	27	19.6	0.82 (d, *J* = 6.44)
5	74.9	-	28	17.6	0.91 (d, *J* = 6.80)
6	73.4	4.80 (d, *J* = 4.7)	1′/1″	173.3, 173.0	-
7	114.0	5.28 (m)	2′/2″	34.6, 34.6	2.25 (t, *J* = 7.5); 2.30 (t, *J* = 7.2)
8	145.6	-	3′/3″	24.9, 25.0	1.60 (m)
9	43.2	2.02 (m)	4′/4″	29.1–29.7	1.23–1.35 (m)
10	37.3	-	5′/5″	29.1–29.7	1.23–1.35 (m)
11	21.9	1.57 (m)	6′/6″	29.1–29.7	1.23–1.35 (m)
12	39.1	1.32 (m); 2.05 (m)	7′/7″	29.1–29.7	1.23–1.35 (m)
13	43.7	-	8′/8″	27.2, 27.2	2.03 (m)
14	54.8	1.91 (m)	9′/9″	127.8, 127.9	5.30–5.40 (m)
15	22.8	1.39 (m); 1.42 (m)	10′/10″	128.0, 128.0	5.30–5.40 (m)
16	27.8	1.72 (m)	11′/11″	25.6, 25.6	2.77 (t, *J* = 6.3)
17	55.9	1.27 (m)	12′/12″	130.0, 130.0	5.30–5.40 (m)
18	12.3	0.57 (s)	13′/13″	130.2, 130.2,	5.30–5.40 (m)
19	18.2	1.06 (s)	14′/14″	27.2, 27.2	2.03 (m)
20	40.4	2.01 (m)	15′/15″	29.1–29.7	1.23–1.35 (m)
21	21.1	1.02 (d, *J* = 6.56)	16′/16″	31.5, 31.9	1.26 (m)
22	135.3	5.19 (m)	17′/17″	22.5. 22.7	1.29 (m)
23	132.1	5.21 (m)	18′/18″	14.0, 14.1	0.88 (m)

**Table 2:** IC_50_ values ^a^ (μM) of compounds **5**–**7** and **9** as inhibitors of TNF-α, IL-6 and NO.

Compounds	TNF-α	IL-6	NO
**5**	78.50 ± 3.72	56.33 ± 6.81	87.31 ± 8.77
**6**	298.50 ± 18.77	-	-
**7**	168.30 ± 9.69	-	-
**9**	62.46 ± 3.18	48.50 ± 6.54	76.16 ± 9.11
Aspirin ^b^	27.08 ± 1.86	28.43 ± 4.46	51.82 ± 8.62

^a^ Data are expressed as mean ± SD; *n* = 5 independent experiments. ^b^ Positive control.

## Data Availability

Not applicable.

## Sample Availability

Samples of the compounds are available from the authors.
